# Narcotic-Free, Over-the-Counter Pain Management After Wide-Awake Hand Surgery

**DOI:** 10.5435/JAAOSGlobal-D-19-00137

**Published:** 2019-11-04

**Authors:** Shafic Sraj

**Affiliations:** From the Department of Orthopaedics, West Virginia University, Morgantown, WV.

## Abstract

Prescribing opioids for postoperative pain has increased steadily, and hand surgery has been no exception. Current hand surgery literature does not describe the efficacy of pain self-management postoperatively. The purpose of this study is to describe our experience with over-the-counter (OTC), narcotic-free, postoperative pain management.

**Methods:**

We have done a retrospective chart review at an academic tertiary-care facility. Patients who underwent soft-tissue hand surgery in an office-based procedure room between January 1, 2018, and March 1, 2019, done using wide-awake local anesthesia only with no tourniquet were included.

**Results:**

Eighty-one continuous patients met the inclusion and exclusion criteria. The procedures included carpal tunnel release, cubital tunnel release, trigger finger release, first dorsal compartment release, dorsal wrist ganglion cyst excision, hand or finger mass excision, percutaneous needle fasciotomy, flexor tendon repair, and extensor tendon repair. Two patients (2.4%) received a prescription for pain medication within 1 day and 4 within 1 week after discharge (total 5.6%). Nine patients were already on chronic narcotics, and four were on short-term narcotics before the surgery date. No other patients requested or received opioid prescriptions after surgery, and no complaints were reported.

**Discussion:**

This study indicates that patients can successfully self-manage their postoperative pain with OTC analgesics. They rarely request prescriptions for pain control after soft-tissue hand surgery. Our findings support current literature suggesting that narcotic prescriptions can be eliminated in select hand and upper extremity procedures and suggest that OTC postoperative pain management is sufficient.

The practice of prescribing opioids for postoperative pain has increased steadily over the past few decades.^1–3^ Hand surgery has not been an exception, and patients have received more opioids than needed.^1,3–5^ Several studies have challenged the necessity of routine prescription of opioids after a variety of hand surgeries.^2–4,6,7^ Current literature suggests that patients undergoing hand surgery consume 10 or fewer doses of analgesics irrespective of what the analgesic is.^2–4,6,7,8,9^ Opioids and non-narcotic analgesics provide equivalent pain control for soft-tissue hand surgery.^6,7^

Current hand surgery literature does not describe the efficacy of pain self-management postoperatively. The purpose of this study is to describe our experience with over-the-counter (OTC), narcotic-free, postoperative pain management in a continuous cohort of 81 patients who underwent soft-tissue hand surgery in an office-based setting over a 14-month period.

## Methods

A retrospective chart review at an academic tertiary-care facility was done after Institutional Review Board approval was granted; patient consent was not required. We included all patients who underwent soft-tissue hand surgery by the author, in an office-based procedure room between January 1, 2018, and March 1, 2019. All procedures were done using wide-awake, local anesthesia only, with no tourniquet (WALANT). We excluded patients who received a prescription of scheduled narcotic pain medications on the day of surgery.

All patients received pain management counseling on three occasions: the first visit during the surgical consent process, just before the procedure before the administration of local anesthesia, and during the procedure itself. The counseling was verbal and was provided by the attending surgeon: Patients were educated that the planned surgery was a minor procedure done under local anesthesia that did not require sedation or other forms of anesthesia. The patients were educated that the procedure is well tolerated by most patients and does not typically require prescription analgesics. The patients were asked to use the OTC analgesics of their choice and to follow the instructions provided on the packages. Acetaminophen and ibuprofen were provided as examples. We did not make any solid recommendation regarding frequency nor dosage other than following label instructions. Patients were scheduled to follow-up within 2 weeks postoperatively and encouraged to contact the surgeon earlier if necessary. Patients were provided with a variety of methods to contact the office, if needed, before their first postoperative visit. They included phone calls, e-mails, and a patient-portal mobile app. We reviewed pain medication prescriptions provided to the patient in the perioperative period, if any, from other providers. This information included all documented prescriptions within the electronic medical record (Hyperspace; Epic, 2018) and data from a multistate database opioid monitoring system. We documented chronic narcotic prescriptions and recent preoperative narcotic prescriptions (within 2 weeks before surgery date) and postoperative narcotic prescriptions (2 weeks after the surgery date). The electronic medical records for all documented communications (phone calls, e-mails, and patient-portal mobile app contact) during the 2-week postoperative period were reviewed.

The primary outcome for this study was patient receipt of narcotic or non-narcotic analgesic prescriptions after they were discharged. Secondary outcomes included timing of those prescriptions and whether there were any documented complaints regarding pain management.

## Results

Eighty-one continuous patients met the inclusion and exclusion criteria. Fifty-two patients were women, and 29 patients were men. The average age on the day of surgery was 53 years (range 17 to 82 years). The total number of procedures was 129, done on 97 upper extremities (50 on the right and 47 on the left), over 84 unique procedure visits. These include multiple procedures including bilateral procedures: 16 procedures were bilateral and included 13 done simultaneously and three done on different dates.

The procedures included carpal tunnel release, cubital tunnel release, trigger finger release, first dorsal compartment release, dorsal wrist ganglion cyst excision, hand or finger mass excision, percutaneous needle fasciotomy, flexor tendon repair, and extensor tendon repair (Table 1). Carpal tunnel release, trigger and first dorsal compartment release, and mass and cyst excisions comprised 85% of the procedures. All patients received a local injection of lidocaine 1% with epinephrine mixed with 8.4% sodium bicarbonate in a 10:1 ratio. No tourniquet was used.

**Table 1:** List of Hand Soft-Tissue Procedures Done

Procedure	Upper Extremities	No. of Procedures
First dorsal compartment release	6	6
Carpal tunnel release	39	39
Cubital tunnel release	3	3
Extensor tendon repair	4	4
Finger mass excision	9	12
Dorsal wrist ganglion cyst excision	4	6
Hand mass excision	2	3
Percutaneous needle fasciotomy	8	8
Trigger finger release	20	29
Wrist mass excision	1	1
Flexor digitorum profundus repair	1	2
Total	97	113

Nine patients (10.7%) were already on chronic analgesics, and four (4.8%) had recent prescriptions for analgesics before the day of surgery. Of the 71 remaining patient visits with no preoperative analgesics, two patients (2.8%) received a prescription for pain medication on the first postoperative day. Two other patients received a prescription for pain medication within 1 week (on the third and fifth days) for a total of four patients (5.6%). It is not clear whether the two delayed analgesic prescriptions were for postoperative pain or other reasons. No other patients requested or received analgesic prescriptions after their surgeries, and no complaints were filed.

## Discussion

The United States consumes 80% of the global opioid supply and 99% of the hydrocodone supply, which makes hydrocodone with acetaminophen the most commonly prescribed medication.^10,11^

Currently, there is no standardized education nor established guidelines for postoperative pain management.^12^ Many physicians, including most surgeons, have not received pain management training in narcotic prescribing. Physicians were asked to address pain as a “fifth vital sign” when, in fact, it was a symptom.^13^ Many surgeons do not know how much to prescribe or how much is actually consumed.^13^ Consequently, surgeons adopted standardized prescription routines without particular attention to the risks of drug dependence or diversion.^2,14^ They also adopted the same amount of narcotics their mentors used to prescribe.^14^

Conversely, many patients report mild, tolerable pain after minor procedures and leave their narcotic prescriptions unfilled.^14^ This trend led several surgical fields to question the necessity of postoperative opioids in outpatient surgery including breast surgery, hernia repair, and laparoscopic cholecystectomy.^7^

These same characteristics equally apply to hand surgery. As many as 59% to 76% of hand surgery patients receive opioid prescriptions^15,16^; in fact, hand surgery patients receive two to five times more opioids than they consume.^1,3–5^ The greatest consumption happens on day 0 and postoperative day 1, and the median for total days of consumption is 2 to 5 days.^2,6,7^

Current hand surgery literature suggests that patients consume 10 or fewer doses of analgesics regardless of what the analgesic is.^2–4,6,7,8,9^ Distal radius fracture patients consume 16 pills or less, on average.^8,9^ Shoulder and elbow surgeries require a mean of 11 to 22 pills for 3 to 5 days.^3^ Patients, however, receive a mean total of 24 pills, frequently in the form of standardized 30-tablet opioid prescriptions.^2,3,14^

Two recent prospective, randomized, double-blinded studies confirmed equivalent pain control between narcotics and non-narcotic analgesics for soft-tissue hand surgery.^6,7^ Ilyas et al.^6^ found no statistical difference in worst daily pain levels or pill consumption after surgery between opioid and nonopioid groups. The patients “overwhelmingly agreed” that 10 pills were enough, although they were not aware what analgesics they received. The authors concluded that, no matter what analgesic is used, no more than five to 10 pills should be prescribed. Weinheimer et al.^7^ showed no difference in the average Visual Analog Score and time until pain-free (a median of 3 to 5 days). Of note, most procedures in these studies were done under local anesthesia^6,7^ which may present a confounding factor. Our surgeries were done exclusively under local anesthesia (WALANT) which facilitates surgeon-patient interaction and intraoperative counseling. It may also avoid any perioperative discomfort due to general anesthesia and tourniquet pain that may negatively affect comfort in the recovery room and pain expectations postoperatively. It remains to be seen whether the choice of local anesthesia affects pain perception and analgesic consumption.

Refill requests are uncommon irrespective of whether an opioid was prescribed initially or not (2% to 7%).^6,7,9^ Patients were satisfied with the amount and potency of the prescribed analgesics regardless of which one it was.^6^ In one study, two of the five patients who requested a stronger pain medication were already on opioids and did not receive any more.^6^ In another study, requests for rescue opioid medication were the same in opioid and nonopioid groups.^7^ Our results are compatible (three of 57 patients, 5.4%) and suggest that nonopioid analgesic prescriptions may not even be necessary.

The probability of long-term opioid use increases sharply in the first days of opioid therapy, particularly after 5 days.^17^ Specific to hand surgery, 13% of opioid-naive patients continue to fill opioid prescriptions beyond 90 days postoperatively.^18^

Sixty-seven to 92% of patients reported unused opioids, which corresponded to 42% to 71% of unused opioid pills. This overprescription creates an opportunity for drug diversion.^1–3,7^ By limiting the frequency and quantity of opioids prescribed, physicians could have a direct and positive effect on the problem of opioid abuse.^7^

Patients anticipate more pain than they actually report postoperatively,^1^ and those who expect more pain have more pain.^19^ For this reason, counseling is a critical part of pain management.^7,14,20^ Patients counseled on the day of and the first day after surgery consume markedly fewer opioid pills yet experience no more pain than those who were not counseled.^20^ Setting patient expectations is a powerful way to decrease the prescribing and use of opioids.^14^ When counseled that a nonopioid regimen was a reasonable alternative, a notable number of patients elected to avoid opioids.^7^ Of 92 patients who met criteria to enroll for a blinded study comparing opioid and nonopioid postoperative pain management, only one patient (1.1%) declined for fear of inadequate postoperative analgesia, whereas 19 patients (21%) declined because they preferred not be exposed to narcotics.^7^ These findings suggest that, given a choice, patients may elect not to receive any narcotics which leads to the question of whether prescribing physicians give patients that choice.

Surgeons choose opioid pain management for various reasons including concerns of undertreatment, avoiding patient complaints, and limiting early requests for refills.^6^ However, this practice comes at a cost.

There are several opportunities for counseling during surgical patient care: on the day the patient decides to have surgery, just before surgery, during surgery (an advantage of wide-awake anesthesia), and immediately after surgery. The discussion should include the concept of pain and being pain-free, as well as expectations regarding analgesic consumption including the need, benefits, and risks of including/excluding opioids, supported by current literature and surgeon experience. The surgeon emphasizes that the goal of pain management does not include being pain-free, but that tolerable pain is expected and adequately controlled.

There are a few limitations to this study. First, there was no control group. We are, however, confident, and this study confirmed that narcotic prescriptions are not necessary for most patients and find it ethically challenging to add a control group just for comparison. We have adopted narcotic-free pain management for all soft-tissue procedures done under WALANT as a standard of care for our practice. Second, some of the patients were already on analgesics before surgery. None of them received extra prescriptions for acute pain. Discounting those patients, 5.6% received postoperative prescriptions. Of those, only half of them (2.8%) received prescriptions within 2 days when pain management is most needed. We are not sure of the reason the delayed two prescriptions were given. Third, we cannot rule out the possibility of consuming previously unused or diverted analgesics.

In review, however, this study confirms that no new prescriptions were provided for the purpose of the procedures, and very few patients requested them. There were no complaints documented.

This study indicates that patient self-management with OTC analgesics was effective and rarely led to requests for rescue analgesic prescriptions. It confirms the current literature regarding the limited need for opioid prescriptions after soft-tissue hand surgery. Eliminating the use of opioid analgesics may be appropriate with adequate patient education and setting expectations and may improve patient satisfaction by reducing short-term side effects; it may also decrease misuse and the overall strain on the healthcare system. This study supports the elimination of opioid prescriptions altogether in select hand surgical procedures, and we have adopted this standard for office-based hand surgery.
